# iTRAQ-Based Proteomics Screen identifies LIPOCALIN-2 (LCN-2) as a potential biomarker for colonic lateral-spreading tumors

**DOI:** 10.1038/srep28600

**Published:** 2016-06-24

**Authors:** Xianfei Wang, Aimin Li, Yubin Guo, Yadong Wang, Xinhua Zhao, Li Xiang, Zelong Han, Yue Li, Wen Xu, Kangmin Zhuang, Qun Yan, Jietao Zhong, Jing Xiong, Side Liu

**Affiliations:** 1 Department of Gastroenterology, Nanfang Hospital, Southern Medical University, Guangzhou, China; 2 Department of Gastroenterology, Affiliated Hospital of North Sichuan Medical College, Nanchong, China; 3 Department of Gastroenterology, Mianyang Central Hospital, Mianyang, China; 4 Department of Gastroenterology, Longgang Central Hospital, Shen Zhen, China

## Abstract

The improvement and implementation of a colonoscopy technique has led to increased detection of laterally spreading tumors (LSTs), which are presumed to constitute an aggressive type of colonic neoplasm. Early diagnosis and treatment of LSTs is clinically challenging. To overcome this problem, we employed iTRAQ to identify LST-specific protein biomarkers potentially involved in LST progression. In this study, we identified 2,001 differentially expressed proteins in LSTs using iTRAQ-based proteomics technology. Lipocalin-2 (LCN-2) was the most up-regulated protein. LSTs expression levels of LCN-2 and matrix metallopeptidase-9 (MMP-9) showed positive correlation with worse pathological grading, and up-regulation of these proteins in LSTs was also reflected in serum. Furthermore, LCN-2 protein overexpression was positively correlated with MMP-9 protein up-regulation in the tumor tissue and serum of LST patients (former *r*_*s*_ = 0.631, *P* = 0.000; latter *r*_*s*_ = 0.815, *P* = 0.000). Our results suggest that LCN-2 constitutes a potential biomarker for LST disease progression and might be a novel therapeutic target in LSTs.

Laterally spreading tumors (LSTs) of the colorectum are a specialized type of flat adenoma, greater than 10 mm in diameter, which typically extend laterally rather than vertically along the luminal wall. LSTs were initially reported by Kudo *et al*.^1^,^2^. Originally, LSTs were only incidentally identified, but with the advent of magnification chromoendoscopy^3^,^4^, LSTs are now regularly detected in approximately 6% of early-stage colorectal cancers^5^,^6^. While LSTs are presumed to constitute an aggressive type of colonic neoplasm, the pathogenesis of this disease is not fully understood. Previous reports mainly focused on the clinicopathological characteristics of LSTs, and only a few studies have reported on their underlying molecular biology.

Based on endoscopic morphology, LSTs are classified into two types and four subtypes: granular type (LST-G) is a distinguished group, which includes the homogeneous G-subtype and nodular mixed G-subtype; and non-granular type (LST-NG) includes the flat elevated NG-subtype and a pseudodepressed NG-subtype^7^,^8^. Previous studies have indicated that LSTs develop into high-grade intraepithelial neoplasia (HIGN) with a frequency ranging from 20.9% to 33.8%^9^,^10^. Although LSTs may be less invasive than other types of polypoid tumors of similar size^11^,^12^, it is evident that LSTs are prone to develop into a deeper submucosal invasive cancer^13^, although there are distinct incidences for such progression with respect to the four different subtypes^14^. Designing rational strategies for dealing with the clinical challenge posed by LSTs has been difficult, primarily due to the lack of insight regarding the mechanisms mediating LSTs pathogenesis. Consequently, there is an urgent need to identify LST-specific protein biomarkers potentially involved in LSTs progression. Furthermore, therapeutic targets for LST-derived metastatic cancer are still elusive. Consequently, enhanced insight into the nature of LST processes constitutes one of the most important open questions in contemporary research on colorectal carcinogenesis.

The above-mentioned considerations prompted us to execute an unbiased approach for identifying LST-specific events in global protein expression. To this end, we employed iTRAQ to profile LSTs and compare results to alternative colonic neoplasms as well as to untransformed colonic tissue. The results identify an unexpected role for lipocalin-2 (LCN-2) and matrix metallopeptidase-9 (MMP-9) as protein biomarkers for LST development and progression.

## Results

### Clinical characteristics of patients with LSTs

The clinical characteristics of the 90 patients suffering from LSTs are summarized in Supplementary Table 1. There were no significant differences with respect to age, gender, or tumor size between LST-G and LST-NG patients (*P* > 0.05). We concluded that out cohort was suitable for indentifying LST-specific protein expression patterns.

### Identification of LST-specific protein expression signatures as revealed by iTRAQ labeling and LC-MS/MS

We applied iTRAQ to a sub-selection of our clinical samples, comparing results to protruded-type colorectal adenomas, small flat adenomas (d < 1 cm), TNM stage I colorectal carcinomas, and the healthy controls. A total of 4,955 proteins were identified from 22,587 individual unique peptides. Of these, 2,001 showed differential expression patterns in the groups compared, and 55 LST-specific events were identified: 14 proteins were specifically up-regulated (Supplementary Table 2) and 41 proteins were specifically down-regulated (Supplementary Table 3).

To provide further understanding of these results, the biological processes and molecular functions of the 2,001 identified proteins were classified according to gene ontology (GO) annotation analysis. Pie charts representing the results obtained for these respective GO categories are shown in Supplementary Figures 1–3. Among these, cellular process, cell part, and protein binding were the most abundant categories in biological process (BP), cellular component (CC) and molecular function (MF), respectively. Furthermore, Kyoto Encyclopedia of Genes and Genomes (KEGG) pathway enrichment analysis revealed that these proteins were mainly involved in metabolic pathways (17.38%), biosynthesis of secondary metabolites (6.09%), focal adhesion (3.67%), pathways in cancer (3.62%), and regulation of actin cytoskeleton (3.57%), suggesting that LSTs are indeed characterized by signal transduction activity defining their specific behavior.

**Figure 1:**
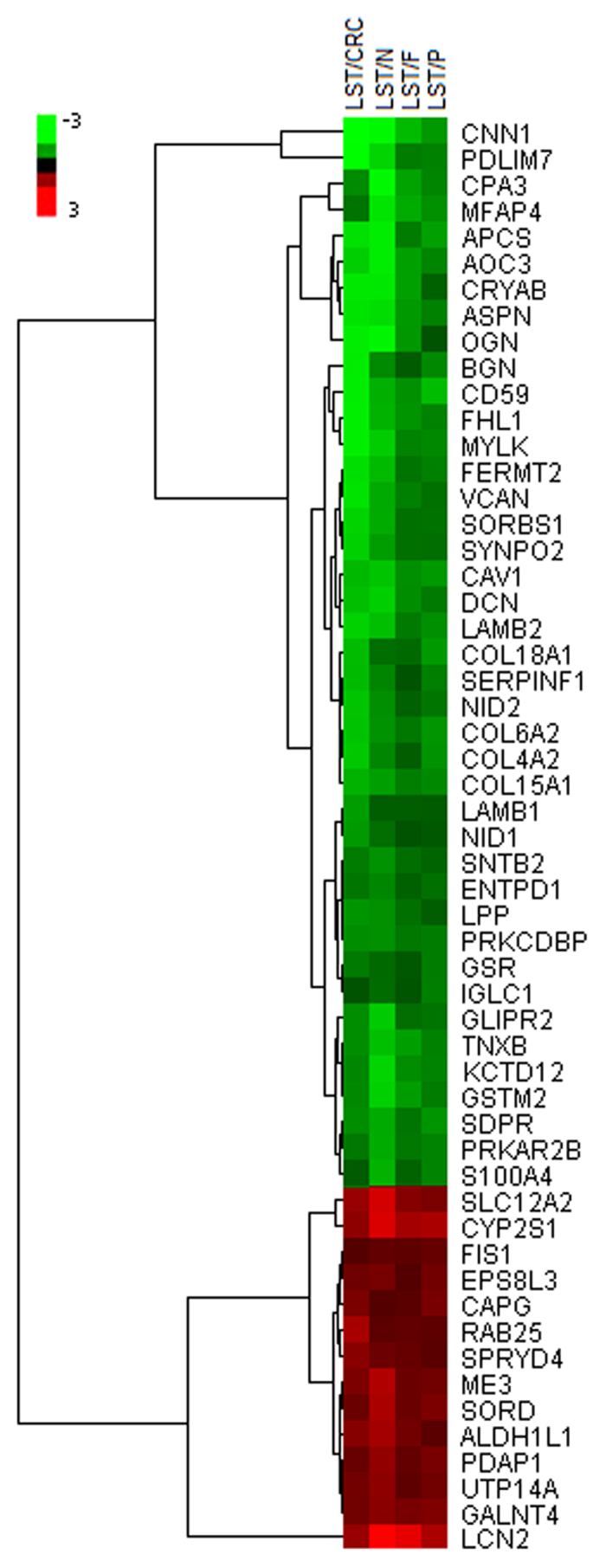
Cluster analysis of 55 differentially expressed proteins between LSTs and other groups. The protein expression levels are shown as colored boxes; red indicates a higher expression level and green indicates a lower expression level. LSTs: laterally spreading tumors, P: protruded-type adenomas, F: small flat adenomas, N: normal controls, CRC: TNM stage I colorectal carcinomas.

Cluster analysis was performed to characterize the specific and unique expression patterns of the 55 differentially expressed proteins, and, as expected, it confirmed significantly altered expression levels of the proteins in LSTs from other groups (Fig. 1). According to the functional annotation, most of the 55 identified proteins were functionally related to specific cell processes, including protein binding, catalytic activity, enzyme regulator activity, and transporter activity.

### Western blot validation

**Figure 2:**
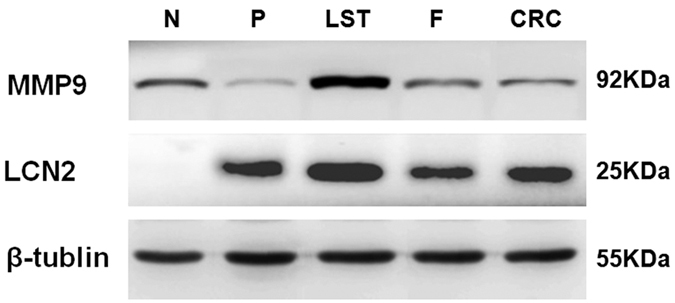
Western blot validation of LCN-2 and MMP-9 in different groups. The expression levels of LCN-2 and MMP-9 were significantly higher in LSTs compared with other groups (*P* < 0.05), which were in line with the iTRAQ results. β-tubulin was used as a loading control. LSTs: laterally spreading tumors, P: protruded-type adenomas, F: small flat adenomas, N: normal controls, CRC: TNM stage I colorectal carcinomas.

We performed Western blot analysis to validate the key proteomic differences identified by iTRAQ analysis. As shown in Fig. 2, the expression levels of LCN-2 and MMP-9 were significantly higher in LSTs compared to other groups (*P* < 0.05, respectively), consistent with the iTRAQ results.

### Immunohistochemistry (IHC) analyses of LCN-2 and MMP-9

**Figure 3:**
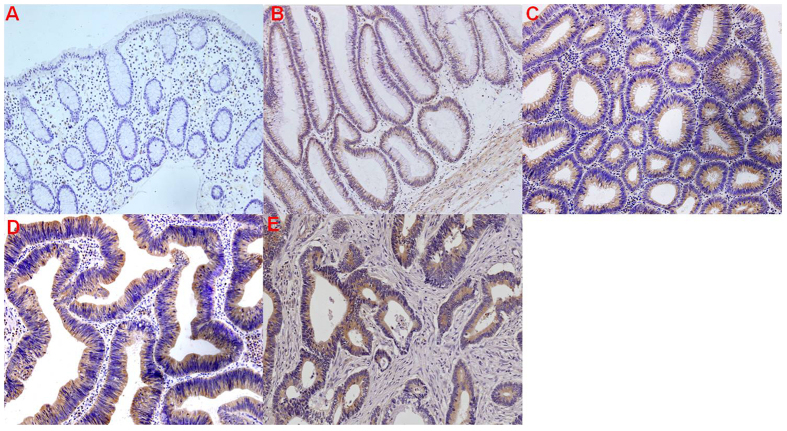
Immunohistochemistry results of LCN-2 expression in the different clinical groups. (**A**) Absence of staining for LCN-2 in the normal colorectal mucosa. LCN-2 immunohistochemical expression was evident in the cytoplasm of the neoplastic cells of LSTs and TNM stage I colorectal carcinomas, and was further increased in LSTs with LST progression. (**B**) Weak LCN-2 staining in low-grade intraepithelial LSTs neoplasia. (**C**) Moderate LCN-2 staining in moderate intraepithelial LSTs neoplasia. (**D**) Marked LCN-2 staining in high-grade intraepithelial LSTs neoplasia. (**E**) Moderate LCN-2 staining in TNM stage I colorectal carcinoma. (original magnification, 200×).

**Figure 4:**
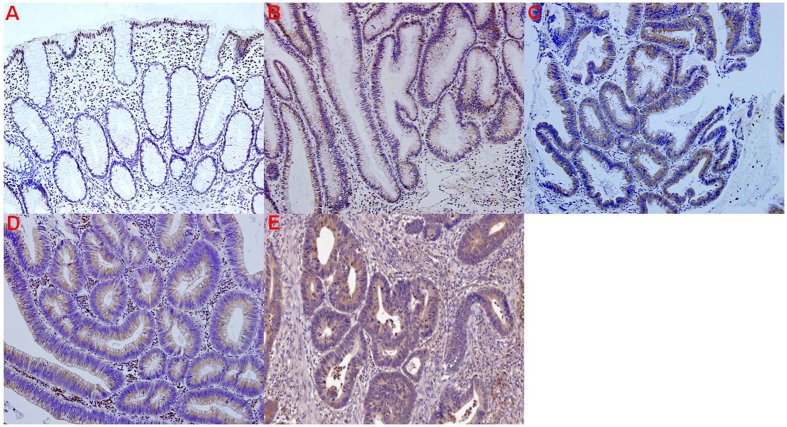
Immunohistochemistry results of MMP-9 in the different clinical groups. (**A**) Absent or faint MMP-9 staining in the normal colorectal mucosa. MMP-9 immunohistochemical expression was present in the cytoplasm of the neoplastic cells of LSTs and TNM stage I colorectal carcinomas, and staining increased in LSTs with LST progression. (**B**) Weak MMP-9 expression in low-grade intraepithelial LSTs neoplasia. (**C**) Moderate MMP-9 expression in moderate intraepithelial LSTs neoplasia. (**D**) Marked MMP-9 expression in high-grade LSTs intraepithelial neoplasia. (**E**) Moderate MMP-9 expression in TNM stage I colorectal carcinoma. (original magnification, 200×).

To further confirm the cellular location of the differentially expressed proteins in LSTs, we selected LCN-2 and MMP-9 for IHC analysis. The specific sites of positive staining for LCN-2 and MMP-9 were cytoplasm in LSTs and TNM stage I colorectal carcinomas, whereas no staining or only faint staining was found in the normal control group. Furthermore, with the increase of pathological grading, the staining intensity of LCN-2 and MMP-9 in neoplastic cells of LSTs gradually increased, and LCN-2 and MMP-9 staining intensity in high-grade intraepithelial LSTs was more intense than in TNM stage I colorectal carcinomas, respectively (Figs. 3 and 4).

Spearman rank correlation analysis between LCN-2 and MMP-9 immunohistochemical scoring and histological grade of LSTs, indicated that the LCN-2 score was positively associated with the MMP-9 score (*r*_*s*_ = 0.631, *P* = 0.000) and the LCN-2 score and MMP-9 score positively correlated with pathological grading (former *r*_*s*_ = 0.943, *P* = 0.000; latter *r*_*s*_ = 0.684, *P* = 0.000). There was no correlation between LCN-2 and MMP-9 immunohistochemical scoring and different types of LSTs based on endoscopic morphology (*P* < 0.05, respectively).

LCN-2 expression is an intrinsic specific property for LST cancer and does not depend on specific recruitment of other hematopoietic or mesenchymal components. However, MMP-9 is closely associated with metastatic processes in general. These findings provide a rational explanation for the aggressive behavior of this tumor. We conclude that LCN-2 and MMP-9 are *bona-fide* markers for disease progression in LSTs.

### Enzyme linked immunosorbent assay (ELISA) for LCN-2 and MMP-9

**Figure 5:**
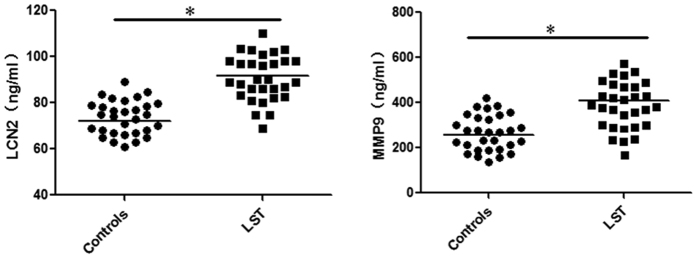
Protein expression of serum LCN-2 and MMP-9 detected by ELISA in the different clinical groups. Increased LCN-2 and MMP-9 serum levels were observed for all LSTs patients compared to healthy controls (*P* < 0.05).

Further confirmation that LSTs are accompanied by increased protein levels of LCN-2 and MMP-9 was provided upon testing serum samples from LSTs patients. When our entire cohort was compared to healthy controls, we found a substantial up-regulation of these proteins in serum from LSTs patients (Fig. 5). It is noteworthy that a significant positive correlation was observed between LCN-2 and MMP-9 serum levels (*r*_*s*_ = 0.815, *P* = 0.000), and that LCN-2 and MMP-9 serum levels had a positive correlation with pathological grading, respectively (former *r*_*s*_ = 0.927, *P* = 0.000; latter *r*_*s*_ = 0.924, *P* = 0.000). These findings suggest that serum levels of these two proteins may be useful for monitoring disease progression. No significant correlations, however, were detected between the expression of LCN-2 and MMP-9 in the serum of LSTs patients and the other clinicopathological parameters of LSTs. Taken together, these results indicate that serum LCN-2 levels correlate with LST disease progression.

## Discussion

Despite their obvious clinical relevance, there is remarkably little known about the molecular properties of LSTs. Here we used an iTRAQ-based proteomics approach to compare protein expression in LSTs compared to clinically relevant control groups. A total of 55 differentially expressed proteins were identified; among them 14 were up-regulated and 41 were down-regulated in LSTs when compared to protruded-type colorectal adenomas, small flat adenomas (d < 1 cm), TNM stage I colorectal carcinomas, and the healthy controls. Bioinformatic analysis revealed that most of these 55 proteins were functionally related to specific cell processes, including protein binding, catalytic activity, enzyme regulator activity, and transporter activity, and our results reveal unique cell biological properties of LSTs. To our knowledge, our study is the first application of iTRAQ-based proteomics technology to identify protein biomarkers that correlate with LSTs pathogenesis.

The finding that LCN-2 is the most up-regulated protein in LSTs compared to other groups is remarkable. Interestingly, serum LCN-2 expression correlates with LSTs disease progression and may thus be useful as a future biomarker to monitor clinical success of LSTs treatment in patients.

LCN-2 is a 25 kDa secreted glycoprotein, also known as neutrophil gelatinase-associated lipocalin (NGAL), belonging to the lipocalin protein family. This gene family performs a variety of functions following ligand binding in a variety of biological processes including cell regulation, proliferation, and differentiation^15^,^16^. Although elevated LCN-2 expression was rarely reported in gastrointestinal cancer before, increased LCN-2 expression had been observed in other malignancies, including breast cancer, pancreatic cancer, and ovarian cancer^17^,^18^.

LCN-2 usually exists as either a monomer, a homodimer, or a heterodimer with MMP-9^19^,^20^. As a member of the MMP family, MMP-9 degrades the basement membranes and extracellular matrix, thus liberating vascular endothelial growth factor (VEGF) from the extracellular matrix enabling angiogenesis, invasion, and metastasis^21^,^22^. Hence, it is tempting to speculate that LCN-2 may participate in the development and progression of cancer by preventing MMP-9 autodegradation^23^,^24^. The notion that MMP-9/LCN-2 complexes, at least *in vitro*, can protect MMP-9 from autodegradation is further supported by observations that LCN-2-overexpressing tumors display increased levels of MMP-9^25^,^26^. However, until such heterodimers are directly detected in LSTs, other possibilities should be kept in mind.

Due to distinct functions of LCN-2 in different cell types, LCN-2 overexpression could increase or suppress tumor cell proliferation, invasion, and metastases in different cancers^27^,^28^. However, little is known about the role of LCN-2 in LSTs. Its association with clinicopathological characteristics and expression of MMP-9 in LSTs has not been reported systematically. Therefore, to further determine the potential biological roles of LCN-2 in LSTs, we detected protein expression levels of LCN-2 and MMP-9 in matched colorectal and serum samples by Western blot, IHC, and ELISA, and then further evaluated the correlation between LCN-2 and MMP-9 as well as clinicopathological features in LSTs.

Western blot results indicate that LCN-2 and MMP-9 expression levels are significantly up-regulated in LSTs compared to other groups (*P* < 0.05), which is in line with the iTRAQ results. To further validate the LCN-2 and MMP-9 protein expression in LSTs tissues, we also performed immunohistochemical analysis. As expected, the normal colon epithelia and stroma were negative for LCN-2 and MMP-9, whereas LCN-2 and MMP-9 showed a strong immunohistochemical reaction in the cytoplasm of the neoplastic cells of LSTs. With the increase in pathological grading, staining intensities of LCN-2 and MMP-9 in neoplastic cells became stronger, and in high-grade intraepithelial LSTs expression was greater than in TNM stage I colorectal carcinomas. Moreover, the two proteins correlated well, in line with the role of these two proteins in the cancerous process. The significance of the observation is further highlighted by our results in patient serum, where absolute levels for both proteins correlated with disease progression but also showed strong correlation with each other. Hence, both proteins appear to be excellent diagnostic markers and are suitable for monitoring disease progression. In addition, more speculatively, the LCN-2-MMP-9 axis may be a novel therapeutic target, as expression levels and correlation to disease progression are unusually strong, suggesting causal links. Further investigation will shed light on the validity of this notion.

With the world-wide application of magnification chromoendoscopy, LST detection rates are rapidly increasing. When LSTs are detected at early stages, endoscopic mucosal resection (EMR) or endoscopic submucosal dissection (ESD) is usually a curative option^29^. Thus, a serum biomarker allowing selection of patients at risk may be exceedingly useful for better screening and prevention of colorectal cancer in general, and for LSTs in particular. Thus, MMP-9/LCN-2 may provide an obvious way forward, especially when used in conjunction for detection^30^.

In conclusion, LCN-2 was the most significantly up-regulated differentially expressed protein in LSTs, while tissue expression levels of LCN-2 and MMP-9 in LSTs positively correlated with pathological grading. Serum increases in LCN-2 and MMP-9 mirrored these effects. Thus, LCN-2 is associated with LSTs disease progression and the function and the potential role of LCN-2 in LSTs needs urgent clarification. In addition, combined serum LCN-2/MMP-9 measurements appear promising for early diagnosis and detection of LSTs disease progression.

## Methods

### Tissue and serum specimens

A total of 160 LSTs were endoscopically collected at NanFang Hospital in GuangZhou, China, between December 2008 and September 2013. For iTRAQ analysis, 30 cases tissue specimens in each group including LSTs, protruded-type colorectal adenomas, small flat adenomas (diameter (d) < 1 cm), TNM stage I colorectal carcinomas, and the normal controls were randomly selected endoscopically, and were promptly frozen in liquid nitrogen and subsequently stored at −80 °C. The correct clinicopathological identification of all samples was confirmed through histopathological evaluation of preoperative endoscopic biopsies performed by an experienced pathologist. In addition, serum samples from the LSTs group and normal control group were collected. Informed written consent for the study was obtained from all enrolled subjects, and the ethical protocols were approved by the Ethics Committee of NanFang Hospital. All the methods were carried out in accordance with the approved guidelines.

### Protein lysis, digestion and labeling with 8-plex iTRAQ reagents

To extract proteins from the specimens, 60 mg of frozen tissue from each group was grinded into powder in liquid nitrogen, and then dissolved in a lysis buffer containing 1 mM phenylmethylsulfonyl fluoride (PMSF), 2 mM ethylenediamine tetraacetic acid (EDTA) and 10 mM dithiothreitol (DTT). The lysate was sonicated with a probe sonicator for 15 min followed by centrifugation at 25,000 x g for 20 min at 4 °C. The supernatant was then collected, and protein concentration was detected using the Bradford method as described previously^31^.

For trypsin-mediated protein digestion, 100 μg protein from each sample was reduced, alkylated, and then digested for 12 h at 37 °C using trypsin (1:20 w/w). The resulting peptides were labeled with iTRAQ 8-plex labeling (Applied Biosystems, Foster City, CA, USA) according to the manufacturer’s instructions. Briefly, iTRAQ tag 113, LSTs-1; 114, protruded-type adenomas-1; 115, small flat adenomas-1; 116, the normal controls; 117, LSTs-2; 118, protruded-type adenomas-2; 119, small flat adenomas-2; 121, TNM stage I colorectal carcinomas. And the iTRAQ-labeled peptides were incubated at room temperature for 2 h.

### SCX chromatography

SCX chromatography was performed using the Shimadzu LC-20AB HPLC pump system according to published methods^32^. Ultimately, 20 fractions were obtained after screening, which were desalted by Strata X C18 column (Phenomenex) and vacuum-dried.

### LC-MS/MS analysis based on Q EXACTIVE

Each fraction was re-suspended in buffer A (2% ACN, 0.1% FA) and centrifuged at 20,000 × g for 10 min to remove the insoluble solid, the final concentration of peptide was about 0.5 μg/μl on average. Then 10 ul supernatant was loaded onto a Shimadzu LC-20AD nano HPLC for separation. The peptides were subjected to nanoelectrospray ionization followed by tandem mass spectrometry (MS/MS) in an QEXACTIVE (Thermo Fisher Scientific, San Jose, CA) coupled online to the HPLC. The process was accomplished as described previously^33^.

### Protein identification and quantification

The Proteome Discoverer software v1.4.0.288 (Thermo Fisher) was applied to process the raw data files and to perform database searches. Protein identification was performed using Mascot search engine (version 2.3.02) against the International Protein Index (IPI) human proteome database (version 3.87; 91464 sequences)^34^.

For protein identification, mass tolerance for precursor ions and fragment ions were set to 10 ppm and 0.5 Da, respectively. False discovery rate (FDR) of both protein and peptide identification were set to be less than 0.01. Confident protein identification involved at least one unique peptide.

### Bioinformatic analysis

To obtain insight into the expression levels of the differentially expressed proteins, the 55 differentially expressed proteins between LSTs and other groups, as identified by initial iTRAQ analysis, were further analyzed by Cluster 3.0 software and the results were visualized using Java Tree View.

GO annotation analysis, including BP, CC and MF of the differentially expressed proteins was performed. Functional annotations of the proteins were made using the Blast2GO program against the non-redundant protein database (NR; NCBI). The KEGG database (http://www.genome.jp/kegg/) and the Cluster of Orthologous Groups of proteins (COG) database (http://www.ncbi.nlm.nih.gov/COG/) were used to classify and group these identified proteins. Network generation and pathway analysis were performed using the Ingenuity Pathway Analysis software package (QIAGEN, Redwood City, CA, USA).

### Western blot

Thirty micrograms of protein samples from LSTs and other groups were separated on a 10% or 12% SDS-PAGE and transferred to nitrocellulose membranes (Millipore). Protein bands were visualized using a conventional enhanced chemiluminescence (ECL) system (GE Healthcare, USA) and quantified with Quantity One (Bio-Rad, USA). Primary antibodies used were anti-LCN-2 antibody (ab23477, 1:500; Abcam), anti-MMP-9 (PAB19095, 1:200; Abnova), and anti-beta tubulin antibody (ab15568, 1:200; Abcam).

### IHC

A total of 90 LSTs, 30 normal tissues, and 30 TNM stage I colorectal carcinomas were subjected to IHC analysis. Briefly, serial consecutive 4-μm tissue sections were cut for subsequent study. Hematoxylin and eosin (H&E) staining was performed according to standard procedures and used for histological verification. IHC staining was performed using EnVision+ Kit (Dako, Denmark). The slices were separately incubated at 4 °C overnight with primary antibodies against LCN-2 (ab23477, 1: 200; Abcam) and MMP-9 (PAB19095, 1:100; Abnova). Based on histological verification by an experienced pathologist, 90 LST cases were classified into intestinal intraepithelial neoplasia, including Mild, Moderate and Severe in this study. For IHC quantitative analysis, scoring of tissue slides was evaluated in a blinded manner by two investigators. The ratio of positively stained cells to all cells in eight random areas at 200-fold magnification was recorded. The total score was then generated based on the average staining intensity and the average percentage of positive cells as previously described^35^.

### Detection of serum LCN-2 and MMP-9 levels by ELISA

To measure the fold changes of LCN-2 and MMP-9, serum was collected from a cohort of patients suffering from LSTs (n = 30) and a normal control group (n = 30). ELISA kits for LCN-2 (RayBiotech, USA) and MMP-9 (Abcam, USA) were used to quantify the concentrations of the serum proteins following the manufacturer’s instructions. ELISA plates were read at 450 nm, and serum protein concentration (for normalization) was determined using Microplate Manager 6 software (Bio-Rad, Inc., Hercules, CA, USA).

### Statistical analysis

Statistical analysis was performed using SPSS 17.0 software (SPSS Inc., Chicago, IL, USA). Data are expressed as means ± standard deviation (SD). One-way analysis of variance (ANOVA) was used to determine significant differences between the groups. The associations between variables were assessed by Spearman’s correlation coefficient. Significance was determined at *P* < 0.05.

## Additional Information

**How to cite this article**: Wang, X. *et al*. iTRAQ-Based Proteomics Screen identifies LIPOCALIN-2 (LCN-2) as a potential biomarker for colonic lateral-spreading tumors. *Sci. Rep*. **6**, 28600; doi: 10.1038/srep28600 (2016).

## Supplementary Material

Supplementary Information

## References

[b1] Kudo S., Kashida H., Nakajima T., Tamura S. & Nakajo K. Endoscopic diagnosis and treatment of early colorectal cancer. World J Surg 21, 694–701 (1997). 9276699 10.1007/s002689900293

[b2] Kudo S. Endoscopic mucosal resection of flat and depressed types of early colorectal cancer. Endoscopy 25, 455–61 (1993). 8261988 10.1055/s-2007-1010367

[b3] Uraoka T. . Endoscopic indications for endoscopic mucosal resection of laterally spreading tumours in the colorectum. Gut 55, 1592–1597 (2006). 16682427 10.1136/gut.2005.087452 PMC1860093

[b4] Hurlstone D. P. . Endoscopic morphological anticipation of submucosal invasion in flat and depressed colorectal lesions: clinical implications and subtype analysis of the Kudo type V pit pattern using high magnificationchromoscopic colonoscopy. Colorectal Dis. 6, 369–375 (2004). 15335372 10.1111/j.1463-1318.2004.00667.x

[b5] Tantau A. I. . Prevalence, histology, endoscopic treatment and long-term follow-up of large colonic polyps and laterally spreading tumors. The Romanian experience. J Gastrointestin Liver Dis. 17, 419–425 (2008). 19104703

[b6] Kudo S. . Colonoscopic diagnosis and management of nonpolypoid early colorectal cancer. World J Surg. 24, 1081–1090 (2000). 11036286 10.1007/s002680010154

[b7] Kudo S. . Nonpolypoidneoplastic lesions of the colorectal mucosa. Gastrointest Endosc 68, S3–47 (2008). 18805238 10.1016/j.gie.2008.07.052

[b8] Kudo S. E., Takemura O. & Ohtsuka K. Flat and depressed types of early colorectal cancers: from East to West. Gastrointest Endosc Clin North Am 18, 581–93 (2008). 10.1016/j.giec.2008.05.013 18674705

[b9] Rotondano G. . The Cooperative Italian FLIN Study Group: prevalence and clinico-pathological features of colorectal laterally spreading tumors. Endoscopy 43, 856–61 (2011). 21826628 10.1055/s-0030-1256639

[b10] Kim B. C. . Clinicopathological differences of laterally spreading tumors of the colorectum according to gross appearance. Endoscopy 43, 100–7 (2011). 21165823 10.1055/s-0030-1256027

[b11] Lambert R. & Tanaka S. Laterally spreading tumors in the colon and rectum. Eur J Gastroenterol Hepatol 24, 1123–1134 (2012). 22732357 10.1097/MEG.0b013e328355e2d9

[b12] Goto H. . Proportion of de novo cancers among colorectal cancers in Japan. Gastroenterology 131, 40–46 (2006). 16831588 10.1053/j.gastro.2006.04.010

[b13] Saito Y. . Endoscopic submucosal dissection (ESD) for colorectal tumors. Dig Endosc. 21, Suppl 1, S7–12 (2009). 19691740 10.1111/j.1443-1661.2009.00870.x

[b14] Tanaka S. . Endoscopic submucosal dissection for colorectal neoplasia: possibility of standardization. Gastrointest Endosc 66, 100–7 (2007). 17591481 10.1016/j.gie.2007.02.032

[b15] Bratt T. Lipocalins and cancer. Biochim Biophys Acta 1482, 318–26 (2000). 11058772 10.1016/s0167-4838(00)00154-0

[b16] Bolignano D. . Neutrophil gelatinase-associated lipocalin (NGAL) in human neoplasias: a new protein enters the scene. Cancer Lett 288, 10–6 (2010). 19540040 10.1016/j.canlet.2009.05.027

[b17] Kubben F. J. . Clinical evidence for a protective role of lipocalin-2 against MMP-9 autodegradation and the impact for gastric cancer. Eur J Cancer 43, 1869–76 (2007). 17604154 10.1016/j.ejca.2007.05.013

[b18] Bauer M. . Neutrophil gelatinase-associated lipocalin (NGAL) is a predictor of poor prognosis in human primary breast cancer. Breast Cancer Res Treat 108, 389–97 (2008). 17554627 10.1007/s10549-007-9619-3

[b19] Kjeldsen L. . Isolation and primary structure of NGAL, a novel protein associated with human neutrophil gelatinase. J Biol Chem 268, 10425–32 (1993). 7683678

[b20] Coles M. . The solution structure and dynamics of human neutrophil gelatinase-associated lipocalin. J Mol Biol 289, 139–57 (1999). 10339412 10.1006/jmbi.1999.2755

[b21] Curran S. & Murray G. I. Matrix metalloproteinases in tumour invasion and metastasis. J Pathol 189, 300–8 (1999). 10547590 10.1002/(SICI)1096-9896(199911)189:3<300::AID-PATH456>3.0.CO;2-C

[b22] Lochter A. . The significance of matrix metalloproteinases during early stages of tumor progression. Ann N Y Acad Sci 857, 180–93 (1998). 9917841 10.1111/j.1749-6632.1998.tb10116.x

[b23] Yan L. . The high molecular weight urinary matrix metalloproteinase (MMP) activity is a complex of gelatinase B/MMP-9 and neutrophil gelatinaseassociated lipocalin (NGAL). Modulation of MMP-9 activity by NGAL. J Biol Chem 276, 37258–65 (2001). 11486009 10.1074/jbc.M106089200

[b24] Fernandez C. A. . The matrix metalloproteinase-9/neutrophil gelatinase-associated lipocalin complex plays a role in breast tumor growth and is present in the urine of breast cancer patients. Clin Cancer Res 11, 5390–5 (2005). 16061852 10.1158/1078-0432.CCR-04-2391

[b25] Tschesche H. . The human neutrophil lipocalin supports the allosteric activation of matrix metalloproteinases. Eur J Biochem 268, 1918–28 (2001). 11277914 10.1046/j.1432-1327.2001.02066.x

[b26] Provatopoulou X. . Circulating levels of matrix metalloproteinase-9 (MMP-9), neutrophil gelatinase-associated lipocalin (NGAL) and their complex MMP-9/NGAL in breast cancer disease. BMC Cancer 9, 390 (2009). 19889214 10.1186/1471-2407-9-390 PMC2775750

[b27] Lee H. J. . Ectopic expression of neutrophil gelatinase-associated lipocalin suppresses the invasion and liver metastasis of colon cancer cells. Int J. Cancer 118, 2490–7 (2006). 16381001 10.1002/ijc.21657

[b28] Hanai J. . Lipocalin 2 diminishes invasiveness and metastasis of Ras-transformed cells. J. Biol. Chem 280, 13641–7 (2005). 15691834 10.1074/jbc.M413047200

[b29] Terasaki M. . Clinical outcomes of endoscopic submucosal dissection and endoscopic mucosal resection for laterally spreading tumors larger than 20 mm. J Gastroenterol Hepatol 27, 734–40 (2012). 22098630 10.1111/j.1440-1746.2011.06977.x

[b30] Narayanan V., Peppelenbosch M. P. & Konstantinov S. R. Human fecal microbiome-based biomarkers for colorectal cancer. Cancer Prev Res (Phila) 7, 1108–11 (2014). 25223933 10.1158/1940-6207.CAPR-14-0273

[b31] Zhao C. . Role of translation by mitochondrial-type ribosomes during sperm capacitation: an analysis based on a proteomic approach. Proteomics 9, 1385–99 (2009). 19253287 10.1002/pmic.200800353

[b32] Zhang T. . Comparative proteomics analysis of placenta from pregnant women with intrahepatic cholestasis of pregnancy. PLoS One 8, e83281 (2013). 24391750 10.1371/journal.pone.0083281 PMC3877025

[b33] Pan H. T. . Differential proteomic analysis of umbilical artery tissue from preeclampsia patients, using iTRAQ isobaric tags and 2D nano LC-MS/MS. J Proteomics 112, 262–73 (2015). 25234496 10.1016/j.jprot.2014.09.006

[b34] Kall L. . Semi-supervised learning for peptide identification from shotgun proteomics datasets. Nat Methods 4, 923–5 (2007). 17952086 10.1038/nmeth1113

[b35] Wang X. F. . Low molecular weight heparin relieves experimental colitis in mice by downregulating IL-1β and inhibiting syndecan-1 shedding in the intestinal mucosa. PLoS One 8, e66397 (2013). 23874391 10.1371/journal.pone.0066397 PMC3715511

