# Supplementary material for: iTRAQ-Based Proteomics Screen identifies LIPOCALIN-2 (LCN-2) as a potential biomarker for colonic lateral-spreading tumors
Source: Sci Rep. 2016 Jun 24;6:28600. doi: 10.1038/srep28600 (PMC4919649; doi:10.1038/srep28600)
Supplement: Supplementary Information [file srep28600-s1.doc]

**iTRAQ-Based Proteomics Screen identifies LIPOCALIN-2 (LCN-2) as a potential biomarker for colonic lateral-spreading tumors**

Xianfei Wang^1,2^, Aimin Li^1^, Yubin Guo^1^, Yadong Wang^1^, Xinhua Zhao^1^, Li Xiang^1,3^, Zelong Han^1^, Yue Li^1^, Wen Xu^1^, Kangmin Zhuang^1^, Qun Yan^1^, Jietao Zhong^1^, Jing Xiong^1^, Side Liu^1^*

Supplementary Table 1. Clinicopathological characteristics of LST patients included in the study. (n = 90)

| **VariableVariables** | **N (%)** |
| --- | --- |
| **Age, years** | 42.68 ± 9.39 |
| **Gender, M/F** | 47/43 |
| **Tumor location** |  |
| Proximal colon | 33 (36.7%) |
| Ileocecus | 5 (5.6%) |
| Ascending colon | 16 (17.8%) |
| Transverse colon | 12 (13.3%) |
| Distal colon | 57 (63.3%) |
| Descending colon | 3 (3.3%) |
| Sigmoid colon | 15 (16.7%) |
| Rectum | 39 (43.3%) |
| **Tumor diameter, mm** | 33.76 ± 8.61 |
| **Histopathological grading** |  |
| Lowgrade intraepithelial neoplasia | 30 |
| Moderate intraepithelial neoplasia | 30 |
| High grade intraepithelial neoplasia | 30 |
| **LST type and subtype** |  |
| Granular | 59 (65.6% ) |
| LST-G-H | 23 (25.6% ) |
| LST-G-NM | 36 (40.0% ) |
| Nongranular | 31 (34.4% ) |
| LST-NG-F | 28 (31.1% ) |
| LST-NG-PD | 3 (3.3% ) |

All data are presented as n (%) or mean ± SD. LST: laterally spreading tumors, LST-GH: homogeneous G-type, LST-G-NM: nodularmixed G-type, LST-NG-F: flat elevated NG-type, LST-NG-PD: pseudo-depressed NG-type.

Supplementary Table 2. Up-regulated differentially expressed proteins in LST (n = 14)

| Accession | Description | LST/F ratio | LST/P Ratio | LST/CRC Ratio | LST/N ratio | Category | Function annotation |
| --- | --- | --- | --- | --- | --- | --- | --- |
| IPI00299547 | LCN-2 | 2.548 | 1.759 | 1.59 | 4.418 | Transcription | Binding transporter_activity |
| IPI00291643 | SPRYD4 | 1.271 | 1.22 | 1.48 | 1.3 | Replication | Ubiquitin mediated proteolysis |
| IPI00216057 | SORD | 1.289 | 1.397 | 1.291 | 1.756 | Amino acid metabolism | Binding catalytic_activity |
| IPI00022649 | SLC12A2 | 1.456 | 1.379 | 1.584 | 2.207 | Metabolism | Transporter_activity |
| IPI00027993 | RAB25 | 1.251 | 1.246 | 1.721 | 1.236 | Metabolism | Binding |
| IPI00003970 | ME3 | 1.294 | 1.33 | 1.391 | 1.89 | Transcription | Metabolic pathways |
| IPI00339297 | GALNT4 | 1.371 | 1.411 | 1.328 | 1.492 | Transcription | Metabolic pathways |
| IPI00181833 | EPS8L3 | 1.206 | 1.338 | 1.311 | 1.363 | Translation | T cell receptor pathway |
| IPI00290553 | ALDH1L1 | 1.337 | 1.225 | 1.464 | 1.69 | Energy production | Binding catalytic_activity |
| IPI00164018 | CYP2S1 | 1.691 | 1.748 | 1.507 | 2.323 | Transport and catabolism | Binding catalytic_activity |
| IPI00013297 | PDAP1 | 1.264 | 1.368 | 1.288 | 1.551 | Protein turnover | Biological regulation |
| IPI00007052 | FIS1 | 1.242 | 1.272 | 1.215 | 1.252 | Translation | Binding |
| IPI00027341 | CAPG | 1.227 | 1.37 | 1.385 | 1.202 | Protein turnover | Biological regulation |
| IPI00107113 | UTP14A | 1.246 | 1.327 | 1.334 | 1.51 | Replication | Binding |

Based on protein abundance. A protein was defined as differentially expressed if there was a ratio in excess of 1.2 and *P* < 0.05,. LST: laterally spreading tumors, P: protruded-type adenomas, F: small flat adenomas, CRC: TNM stage I colorectal carcinomas, N: normal controls.

Supplementary Table 3. Down-regulated differentially expressed proteins in LST (n = 41)

| Accession | Description | LST/F ratio | LST/P ratio | LST/CRC ratio | LST/N ratio | Category | Function annotation |
| --- | --- | --- | --- | --- | --- | --- | --- |
| IPI0017354 | SYNPO2 | 0.754 | 0.762 | 0.465 | 0.611 | Cytoskeleton | actin binding |
| IPI00056334 | PRKCDBP | 0.731 | 0.716 | 0.664 | 0.65 | Coenzyme metabolism | protein binding |
| IPI00006114 | SERPINF1 | 0.83 | 0.712 | 0.527 | 0.688 0.688 | Protein turnover | protein-arginine deiminase  activity |
| IPI00219067 | GSTM2 | 0.607 | 0.717 | 0.679 | 0.462 | Energy production | glutathione transferase activity |
| IPI00009236 | CAV1 | 0.654 | 0.627 | 0.531 | 0.498 | Energy production | kinase binding |
| IPI00009802 | VCAN | 0.705 | 0.751 | 0.404 | 0.571 | Lipid metabolism | calcium ion binding |
| IPI00554752 | PRKAR2B | 0.73 | 0.693 | 0.729 | 0.564 | Signal transduction | cAMP binding |
| IPI00026944 | NID1 | 0.83 | 0.819 | 0.627 | 0.763 | Inorganic metabolism | collagen binding |
| IPI00304840 | COL6A2 | 0.731 | 0.637 | 0.501 | 0.647 | Cell motility | extracellular matrix structural  constituent |
| IPI00418431 | ASPN | 0.624 | 0.699 | 0.403 | 0.422 | Cell division | protein binding |
| IPI00021369 | CRYAB | 0.601 | 0.798 | 0.382 | 0.386 | Amino acid metabolism | Biological regulation |
| IPI00009505 | SNTB2 | 0.76 | 0.786 | 0.719 | 0.644 | Protein turnover | actin binding |
| IPI00012119 | DCN | 0.662 | 0.724 | 0.513 | 0.469 | Chaperones | extracellular matrix binding |
| IPI00295414 | COL15A1 | 0.714 | 0.677 | 0.546 | 0.604 | Carbohydratetransport trrttransportmetabolism metabolism metabolism | extracellular matrix structural  constituent |
| IPI00032313 | S100A4 | 0.789 | 0.697 | 0.814 | 0.547 | Cytoskeleton | calcium ion binding |
| IPI00022792 | MFAP4 | 0.567 | 0.65 | 0.743 | 0.394 | Signal transduction | receptor binding |
| IPI00023122 | PDLIM7 | 0.715 | 0.699 | 0.223 | 0.445 | Cytoskeleton | protein binding |
| IPI00025465 | OGN | 0.629 | 0.83 | 0.36 | 0.314 | Lipid metabolism | protein binding |
| IPI00296922 | LAMB2 | 0.723 | 0.654 | 0.457 | 0.509 | Energy production | integrin binding |
| IPI00005809 | SDPR | 0.737 | 0.638 | 0.66 | 0.571 | Cytoskeleton | protein binding |
| IPI00010554 | SORBS1 | 0.755 | 0.749 | 0.452 | 0.565 | Cytoskeleton | protein kinase binding |
| IPI00022391 | APCS | 0.717 | 0.598 | 0.412 | 0.364 | Signal transduction | carbohydrate binding |
| IPI00791032 | MYLK | 0.695 | 0.678 | 0.346 | 0.477 | Replication | actin binding |
| IPI00306322 | COL4A2 | 0.805 | 0.645 | 0.49 | 0.681 | Coenzyme metabolism | protein binding |
| IPI00022822 | COL18A1 | 0.771 | 0.621 | 0.523 | 0.761 | Amino acid metabolism | protein binding |
| IPI00025276 | TNXB | 0.605 | 0.702 | 0.662 | 0.509 | Protein turnover | ATPase activity |
| IPI00021264 | CNN1 | 0.52 | 0.622 | 0.131 | 0.285 | Cytoskeleton | actin binding |
| IPI00004457 | AOC3 | 0.598 | 0.687 | 0.469 | 0.363 | Metabolites metabolism | primary amine oxidase activity |
| IPI00719373 | IGLC1 | 0.823 | 0.71 | 0.83 | 0.768 | Signal transduction | cAMP binding |
| IPI00014398 | FHL1 | 0.642 | 0.692 | 0.353 | 0.543 | Inorganic metabolism | protein binding |
| IPI00011302 | CD59 | 0.651 | 0.516 | 0.359 | 0.553 | Inorganic metabolism | protein binding |
| IPI00009829 | CPA3 | 0.588 | 0.691 | 0.667 | 0.281 | Amino acid metabolism | metallocarboxypeptidase activity |
| IPI00220852 | ENTPD1 | 0.8 | 0.755 | 0.735 | 0.683 | Carbohydrate transport | ATP binding |
| IPI00060715 | KCTD12 | 0.659 | 0.697 | 0.682 | 0.449 | Cell motility | protein binding |
| IPI00010790 | BGN | 0.811 | 0.676 | 0.367 | 0.671 | Amino acid metabolism | extracellular matrix binding |
| IPI00013976 | LAMB1 | 0.802 | 0.811 | 0.607 | 0.807 | Signal transduction | glycosphingolipid binding |
| IPI00016862 | GSR | 0.816 | 0.716 | 0.723 | 0.77 | Cytoskeleton | electron carrier activity |
| IPI00023704 | LPP | 0.755 | 0.803 | 0.64 | 0.652 | Cytoskeleton | protein binding |
| IPI00007067 | GLIPR2 | 0.76 | 0.739 | 0.661 | 0.481 | Amino acid metabolism | protein binding |
| IPI00028908 | NID2 | 0.797 | 0.736 | 0.498 | 0.658 | Energy production | collagen binding |
| IPI00000856 | FERMT2 | 0.739 | 0.705 | 0.419 | 0.526 | Chaperones | phospholipid binding |

Based on protein abundance. A protein was defined as differentially expressed if there was a ratio in excess of 1.2 and *P* < 0.05. LST: laterally spreading tumors, P: protruded-type adenomas, F: small flat adenomas, CRC: TNM stage I colorectal carcinomas, N: normal controls.

**
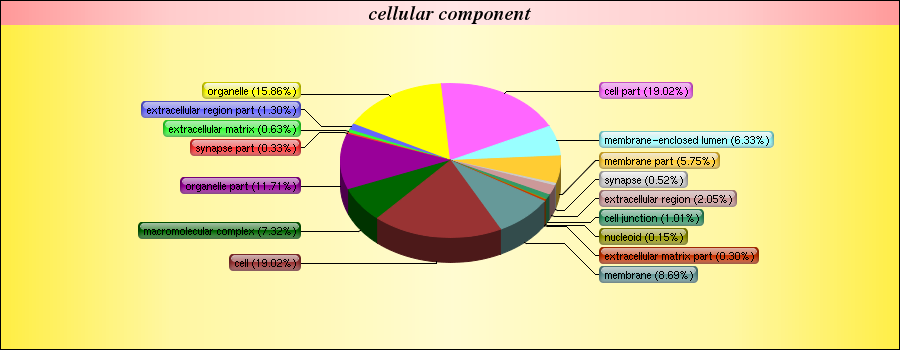
**

Supplementary Figure 1. Pie chart depicting the results of the cellular component analysis of the 2,001 differentially expressed proteins in the four groups.


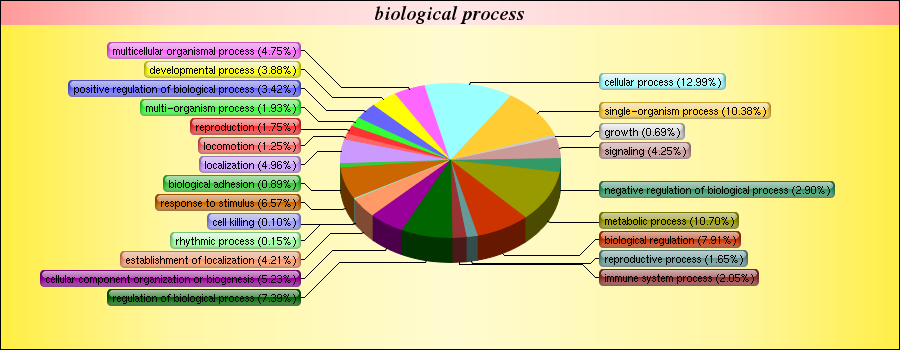


Supplementary Figure 2. Pie chart depicting the results of the biological process analysis of the 2,001 differentially expressed proteins in the four groups.


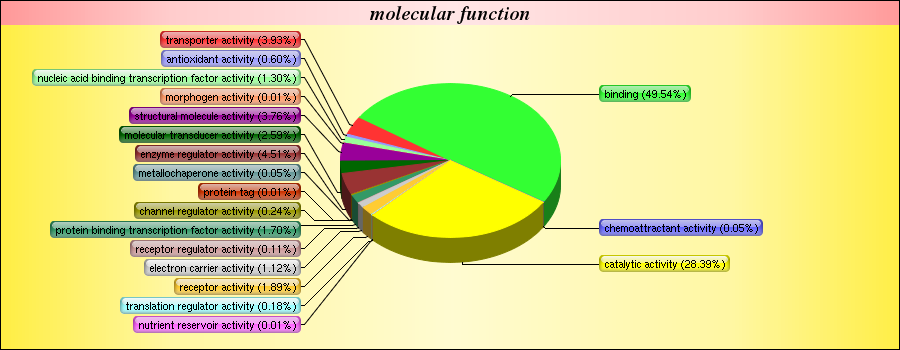


Supplementary Figure 3. Pie chart depicting the results of the molecular function analysis of the 2,001 differentially expressed proteins in the four groups.
